# Integrated analysis from multicentre studies identities m7G-related lncRNA-derived molecular subtypes and risk stratification systems for gastric cancer

**DOI:** 10.3389/fimmu.2023.1096488

**Published:** 2023-03-02

**Authors:** Mingwei Ma, Jie Li, Ziyang Zeng, Zicheng Zheng, Weiming Kang

**Affiliations:** Department of General Surgery, Peking Union Medical College Hospital, Chinese Academy of Medical Sciences and Peking Union Medical College, Beijing, China

**Keywords:** gastric cancer, m7G modification, lncRNA, immunotherapy, prognosis

## Abstract

**Introduction:**

Gastric cancer (GC) is the fourth leading cause of cancer death worldwide. Due to the lack of effective chemotherapy methods for advanced gastric cancer and poor prognosis, the emergence of immunotherapy has brought new hope to gastric cancer. Further research is needed to improve the response rate to immunotherapy and identify the populations with potential benefits of immunotherapy. It is unclear whether m7G-related lncRNAs influence tumour immunity and the prognosis of immunotherapy.

**Methods:**

This study evaluated 29 types of immune cells and immune functions in gastric cancer patients, and m7G-related lncRNAs and their molecular subtypes were identified. In addition, we also studied the biological function characteristics of m7G-related lncRNA molecular subtypes. Finally, the patient's risk score was calculated based on m7G-related lncRNAs, and a nomogram of staging and risk groups was established to predict the prognosis. For experimental verification, RT–qPCR were preformed from the native cohort.

**Results:**

After identifying m7G-related lncRNAs and their molecular subtypes, we found three molecular subtypes, the B subtype had the highest level of infiltration, and the B subtype may benefit more from immunotherapy. We divided GC patients into two regulator subtypes based on biological function. The two subtypes have significant immunological differences and can be used to judge ICI treatment. We established a risk score formula based on five lncRNAs, including LINC00924, LINC00944, LINC00865, LINC00702, and ZFAS1. Patients with poor prognoses were closely related to patients in the high-risk group. After comprehensive analysis of different risk groups, the efficacy of the high-risk group on bleomycin, cisplatin, docetaxel, doxorubicin and etoposide was better than that of the low-risk group, suggesting that risk subgroups based on risk scores play a guiding role in chemotherapy and that the high-risk group may benefit more from immunotherapy. RT–qPCR results showed that LINC00924, LINC00944, and LINC00865 were highly expressed in tumour tissues, while LINC00702 and ZFAS1 were expressed at low levels in tumour tissues.

**Discussion:**

In conclusion, we were the first to discover that m7G-related lncRNAs play a vital role in the tumour immune microenvironment of gastric cancer, and a risk prediction model was established to identify patients with potential benefits from immunotherapy and predict the prognosis of GC patients.

## Introduction

Gastric cancer (GC) is currently the fifth most common malignant tumour globally (1). Due to the lack of specific symptoms in the early stage of GC, many patients are diagnosed at an advanced stage with a 5-year survival rate of less than 30% (2–4). The resection of advanced GC is not very effective due to poor prognosis, and there is a lack of effective treatment. In recent years, the emergence of immunotherapy seems to have brought hope to patients with advanced GC, but the effective response rate is low. Therefore, it is urgently needed to study the mechanisms and methods to improve the response rate to immunotherapy.

Tumour immunotherapy is widely used clinically as the fourth treatment method after surgery, radiotherapy and chemotherapy. It can inhibit and kill tumour cells by stimulating or mobilizing the immune system and enhancing the antitumor immunity of the tumour microenvironment. TILs are a major component of tumour-infiltrating immune cells, consisting of T cells, B cells, and NK cells, and have been reported to affect cancer progression and response to immunotherapy (5, 6). CD57+ is a marker of NK cells; similar to CD8+ T cells, CD57+ NK cells directly eliminate tumour cells and exhibit antitumor immunity (7). Studies have shown that although tumour cells have developed various abilities to evade the recognition of CD8+ T cells, they still cannot escape the attack of NK cells (8). A growing body of research has suggested that NK cells play an essential role in the development of GC. Saitto et al. found that the frequency of NK cell apoptosis in GC patients was significantly higher than that in normal tissues, and its frequency was related to the progression of GC (9). CD4+ T cells include all subgroups of helper T cells and regulatory T cells and participate in antitumor cellular and humoral immunity by secreting a variety of interleukins (10). A high ratio is considered to be an effective predictor of postoperative prognosis (11), while a high proportion of circulating Th17 and Th22 cells is associated with tumour progression and poor prognosis in GC (12). The above studies have shown that different infiltrating immune cells and their ratios are closely related to the prognosis of GC.

Epigenetics is a phenomenon in which the gene sequence does not change, but the level of gene expression and modification can produce heritable changes. Among them, the epigenetic methylation modification represented by m7G has attracted widespread attention. During transcription initiation, m7G is cotranscribed onto the 5’ cap (13). This essential cap modification stabilizes transcripts, prevents exonucleolytic degradation, and regulates nearly every step of the mRNA life cycle, including transcription elongation, premRNA splicing, polyadenylation, nuclear export, and translation. As part of the cap structure, m7G is also present inside tRNA and rRNA (14), and internal m7G modifications affect RNA processing and function and are thought to be involved in human diseases. Similar to mRNA in structure, lncRNA also has a 5’ end cap and 3’ end polyadenylic acid tail and splicing phenomenon, but it lacks an obvious open reading frame and does not have a protein-coding function. It regulates the expression levels of genes at various levels, such as transcription and posttranscription, and is widely involved in various physiological and pathological processes in the body (15). Studies have shown that lncRNAs also have important regulatory effects on immune cells (16). CD8+ T cells express many lymphocyte-specific lncRNAs, which are dynamically regulated during cell differentiation or activation states (17). Microarray analysis of mouse spleen naive T cells and memory CD8+ T-cell lncRNA expression profiles showed that nearly one hundred lncRNAs are only expressed in specific tissues or cells (17). The lncRNA Tmevpg1 is expressed in both peripheral blood NK cells and T cells, and its expression is inhibited when stimulated and activated by IFN-γ (18). The above results suggest that lncRNAs play critical regulatory roles in immune cell differentiation and activation. Changes in epigenetic modification-related lncRNAs can be used by tumour cells to disrupt immunogenicity and immune recognition mechanisms, thereby obtaining an immune escape phenotype (19–22). In addition, epigenetic silencing affects almost all antigen processing and presentation processes (23). The vital role of epigenetic modifications in tumour immune escape has laid a solid theoretical foundation for using epigenetic modifiers to improve the immune targeting of tumour cells. However, whether m7G methylation modification affects tumour immunity is still unclear.

To gain a deeper understanding of the mechanisms of gastric carcinogenesis, data from 3 independent GC cohorts from TCGA and GEO were included and analysed. Twenty-nine types of immune cells and immune functions in GC samples were evaluated, and m7G-related lncRNAs and their molecular subtypes were identified. The results suggested that the B subtype may benefit more from immunotherapy; according to the differential mRNAs related to molecular subtypes, GC patients were divided into two subtypes of regulators. In addition, the patient’s risk score was calculated based on m7G-related lncRNAs, resulting in a risk score formula based on five lncRNAs, including LINC00924, LINC00944, LINC00865, LINC00702, and ZFAS1. Finally, the above data were experimentally verified. RT–qPCR results showed that LINC00924, LINC00944, and LINC00865 were highly expressed in tumour tissues, while LINC00702 and ZFAS1 were expressed at low levels in tumour tissues. Our study is the first to indicate that m7G-related lncRNAs play a significant role in the tumour immunity of GC.

## Materials and methods

### Datasets and preprocessing

Our study included 3 independent cohorts from the TCGA and GEO databases. Samples were processed according to the following criteria: (1) primary GC; (2) complete gene expression profiles and survival information; and (3) no chemotherapy or radiotherapy before surgery.

The final GSE66229 cohort included 300 patients, the GSE15459 cohort included 192 patients, and the TCGA cohort included 370 patients. In addition, copy number variation (CNV) and somatic mutation data of GC were downloaded from the TCGA database.

On the GDC website (https://portal.gdc.cancer.gov/), we were able to access RNA sequencing data of gene expression (FPKM values) and clinical information in the TCGA dataset. Transcripts per kilobase million (TPM) were converted from FPKM. Data from the GEO database were annotated from the Affymetrix platform (GPL570). For GSE66229 and GSE15459 microarrays, datasets and clinical information were accessed directly from the GEO website(http://www.ncbi.nlm.nih.gov/geo). The ComBat algorithm was used to eliminate the batch effect of the TCGA and GEO databases in the ‘sva’ package, and the above three cohorts were integrated to establish a meta cohort.

Principal component analysis (PCA) before and after batch correction is shown in **Figure S1**. The GENCODE database (GRCh38 version) was used for lncRNA and mRNA annotation. In addition, taking the intersection of the two platforms (Illumina and GPL570), 1084 lncRNAs and 16192 mRNAs were finally retained for subsequent analysis. M7G-related genes from the literature (24) and related gene sets were obtained from GOMF_M7G_5_PPPN_DIPHOSPHATASE_ACTIVITY and GOMF_RNA_CAP_BINDING GOMF_RNA_7_METHYLGUANOSINE_CAP_BINDING. Twenty-four m7G-related genes were annotated in the final meta-dataset: DCP2, IFIT5, EIF3D, EIF4G3, NSUN2, GEMIN5, AGO2, NUDT10, EIF4E, EIF4E2, NCBP2, NUDT11, NUDT3, NCBP1, METTL1, LARP1, NUDT4, EIF4E3, SNUPN, WDR4, LSM1, NUDT16, DCPS, and CYFIP1. Spearman correlation analysis (*p < 0.001*, correlation coefficient > 0.3) was performed on all lncRNAs and 24 m7G-related genes in the meta cohort. Finally, 123 m7G-related lncRNAs were screened for subsequent bioinformatic analysis.

### Assessment of the immune microenvironment

The ssGSEA score (xi) was calculated for each GC sample (i) using the ssGSEA algorithm and transformed using the formula xi=(xi-xmin)/(xmax-xmin), where xmax and xmin represent the maximum and minimum values of ssGSEA scores, respectively. Scores of 29 immune cells and immune functions in GC samples were calculated, and heatmap visualization was performed using the heatmap package. Additionally, the ESTIMATE algorithm identifies specific features associated with stromal scores, immune cell infiltration and tumour purity. Differences in risk grouping across molecular subtypes were compared using the Kruskal–Wallis test.

### Unsupervised clustering

Unsupervised consensus clustering analysis was performed based on the expression levels of m7G-related lncRNAs. Principal component analysis (PCA) was used to determine whether each subtype was relatively independent of the other subtypes. The number of clusters was determined using the R package “conensusClusterPlus”, and 100 replicates were performed with pltem=0.8 to verify the stability of the subtypes. Kaplan–Meier curves were used to assess the overall time to survival (OS) of patients with different GC in the dataset, and log-rank tests were performed. The ability of molecular subtypes or risk groups to discriminate patients was determined using PCA with a dimensionality reduction method.

### Construction of the risk score model

In the batch-adjusted GEO cohort, univariate Cox regression analysis was used to identify m7G-related lncRNAs associated with prognosis *(p < 0.001*). Subsequently, the least absolute shrinkage and selection operator (LASSO) model was used to remove redundant genes, a risk model was constructed based on the coefficients of multivariate Cox regression, and the TCGA cohort was used as the validation set to test the predictive efficacy. ROC curve analysis was performed using the timeROC software package. Independent prognostic factors identified by multivariate Cox regression were identified to construct prognostic nomograms, and ROC curves were used for calibration curve validation.

### Enrichment analysis

GSVA was used to assess differences in biological pathways between subtypes. Gene Ontology (GO) was used to annotate the biological processes of genes, molecular functions and cellular components. Differential genes between different subtypes were analysed (*p<0.05*), and then the overlapping genes among the three groups were analysed by GO and KEGG using the ‘clusterProfiler’ package. In addition, c2.cp.kegg.v7.0.symbols.gmt was used as a reference gene set, and FDR < 0.05 was the screening threshold.

### Drug sensitivity analysis

IC50 was calculated using the ‘prophetic’ package in R software, and chemotherapeutic drugs were obtained from the genome of the Drug Sensitivity in Cancer (GDSC) database.

### Real-time quantitative PCR

Twenty pairs of GC and paired adjacent tissues were obtained from patients with advanced GC who underwent radical gastrectomy in our hospital from 2021-2022, and TRIzol ^®^ (1 mL) was used to isolate total RNA from tumour and adjacent tissues (200 mg) in the validation dataset. Complementary DNA (cDNA) was created using reverse transcriptase from avian medulloblastoma virus and random primers according to TAKARA’s instructions. SYBR Premix Ex Taq II (Takara, Shiga, Japan) was used for amplification of cDNA, and the process proceeded at 37˚C for 15 min. Then we used SYBR Premix EX TaqTM II (Takara Biotechnology, Dalian, China) on a Bio-Rad IQ5 assay system (Bio-Rad Laboratories, U.S.A.) to determine the mRNA expression levels. The PCR conditions were as follows: predenaturation at 95˚C for 30 s, denaturation at 95˚C for 5 s, and annealing at 60˚C for 30 s, and the complete synthesis progress was 40 cycles. Beta-actin was utilized as an internal reference to normalize the mRNA expression levels of the target genes. and the data were analysed using the 2^-ΔΔCT^ value. Primer sequences are from the Getprime database (https://gecftools.epfl.ch/getprime). All primers were tested for efficiency, and only an amplification efficiency between 90% and 110% was used. **Supplementary File 1** shows the primer sequences.

### Statistical analysis

Spearman correlation analysis was applied to calculate correlation coefficients between the abundance of immune cells and the expression level of m7G-related lncRNAs. The χ2 test was used for associations between categorical covariates. Based on the correlation of the risk score with patient prognosis, the optimal cutoff value for each dataset subset was defined using the “survminer” R package. This value divided patients into high and low risk score subgroups. The log-rank statistic is used to reduce batch effects of calculations. OS plots were drawn by the Kaplan-Meier method and the log-rank test was applied to identify statistical differences. And unpaired T test was applied to identify statistical differences in RT-PCR experiments. P<0.05 was considered statistically significant.

### Ethical statement

The study involving the usage of patient tissues was performed in accordance with the Declaration of Helsinki and was approved by the Ethics Committee of Peking Union Medical College Hospital (approval No. 001933). All of the patients were given and accepted an informed consent form prior to their enrollment.

## Results

### Landscape of m7G regulators in GC

**Figure 1:**
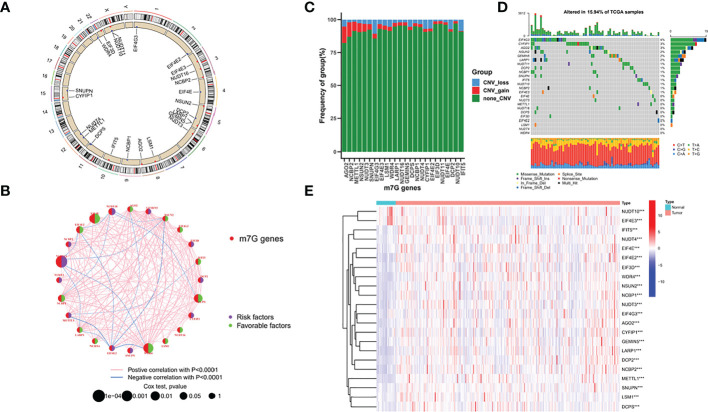
Landscape of m7G regulators in GC. **(A)** chromosomal locations of the 24 m7G regulators **(B)** prognosis and correlation landscape of regulators **(C)** Frequency of group in m7G genes **(D)** mutation frequency of TCGA samples **(E)** differential analysis of the expression of the m7G regulator.

**Figure 1A** shows the chromosomal locations of the 24 m7G regulators that can be annotated. **Figure 1B** shows the prognosis and correlation landscape of regulators. Most of the regulators are closely related, and 14 regulators can be used as indicators of the prognosis of GC patients (**Supplementary Figure 2**). Additionally, we found that AGO2 had the highest frequency of amplification, while EIF4G3 had the highest frequency of deletion (**Figure 1C**). Interestingly, EIF4G3 also had the highest mutation frequency in the samples (**Figure 1D**). Additionally, according to differential analysis of the expression of the m7G regulator in different samples, the results showed that except for NUDT10 and EIF4E3, which were upregulated in normal samples, most genes were upregulated in tumour samples (**Figure 1E**).

### Identification of m7G-associated lncRNAs and their molecular isoforms

**Figure 2:**
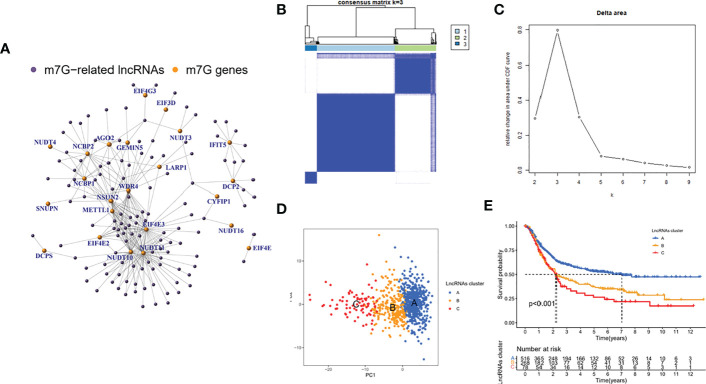
Identification of m7G-associated lncRNAs and their molecular isoforms. **(A)** correlation analysis **(B, C)** consensus cluster and curve **(D)** PCA analysis **(E)** survival analysis.

In the meta cohort, correlation analysis was performed using the abovementioned 24 m7G regulators and all annotable lncRNAs, and a total of 123 m7G lncRNAs were identified (**Figure 2A**). According to the CDF consensus score of the curve, k = 3 was the best (**Figures 2B, C**). PCA demonstrated that the molecular typing based on 123 m7G regulators had significant heterogeneity (**Figure 2D**). In addition, survival analysis showed that type C had the worst prognosis among these three molecular classifications, while type A had the best prognosis (**Figure 2E**).

### Immune characterization of m7G-associated lncRNA molecular subtypes

**Figure 3:**
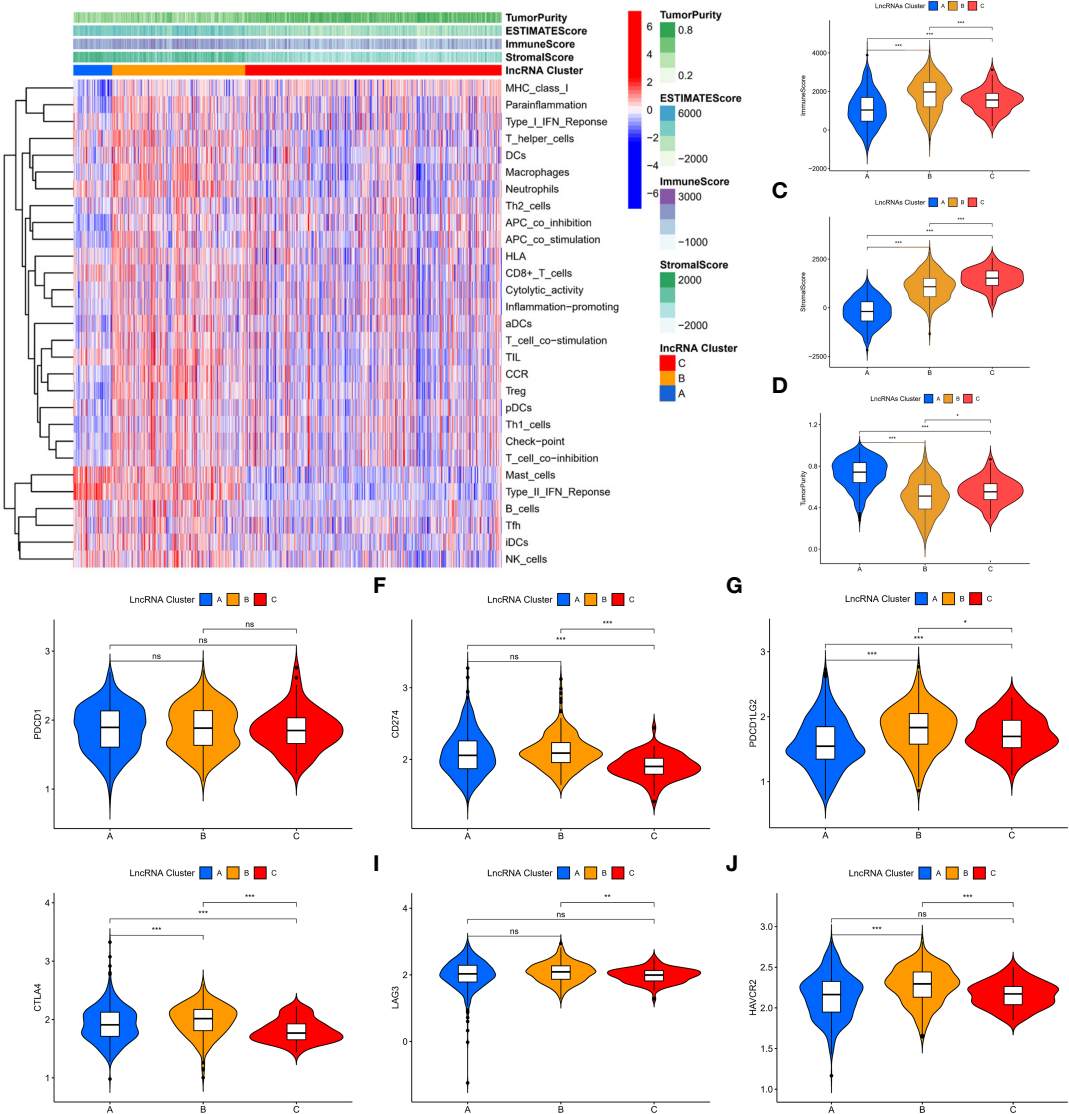
Immune characterization of m7G-associated lncRNA molecular subtypes. **(A)** immune infiltration level **(B-D)** assessment of the stromal scores of the three subtypes, immune scores and tumor purity **(E-J)** expression of six immune checkpoint genes. *P < 0.05, **P < 0.01, ***P < 0.001, ns: no significance.

Among the three molecular subtypes, type B had the highest degree of infiltration, followed by type C and type A (**Figure 3A**). In contrast, type B tumours had the lowest purity, and type A tumours had the highest purity (type A > type C > type B) (**Figures 3B–D**). Finally, we detected the expression of six immune checkpoint genes (i.e., PDCD1, CTLA4, HAVCR2, LAG3, CD274, PDCD1LG2) and found that the mRNA levels of some immune checkpoints in subtype B were higher than those in other types, which may suggest that this subtype may benefit more from immunotherapy (**Figures 3E–J**).

### Biological functional characterization of m7G-associated lncRNA molecular isoforms

**Figure 4:**
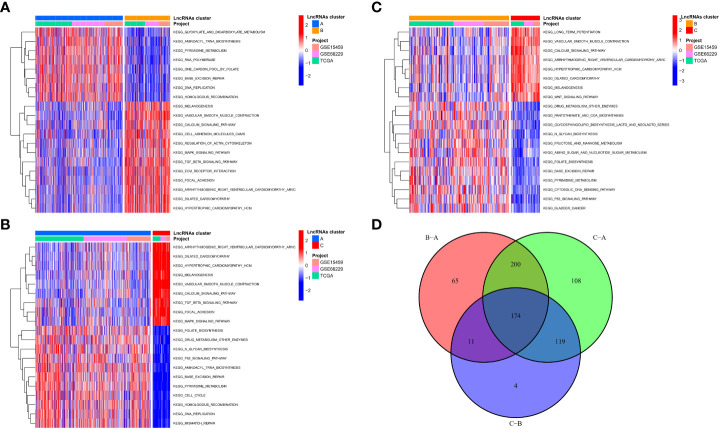
Biological functional characterization of m7G-associated lncRNA molecular isoforms. **(A-C)** GSVA to study changes in biological processes between different subtypes **(D)** 174 mRNAs were considered to be regulator genes of molecular subtypes.

To explore the reasons for the different survival states and immune landscapes, we used GSVA to study changes in biological processes between different subtypes. The results show that compared with type B, type A has more activated pathways, such as KEGG_GLYOXYLATE_AND_DICARBOXYLATE_METABOLISM, KEGG_AMINOACYL_TRNA_BIOSYNTHESIS, KEGG_PYRIMIDINE_METABOLISM, etc.

(**Figure 4A**). Compared with type C, the type A has more active channels, such as

KEGG_ARRHYTHMOGENIC_RIGHT_VENTRICULAR_CARDIOMYOPATHY_ARVC, KEGG_DILATED_CARDIOMYOPATHY, etc. (**Figure 4B**), etc. Type B has more activated pathways, such as KEGG_DRUG_METABOLISM_OTHER_ENZYMES, KEGG_PANTOTHENATE_AND_COA_BIOSYNTHESIS, KEGG_GLYCOSPHINGOLIPID_BIOSYNTHESIS_LACTO_AND_NEOLACTO_SERIE, etc. (**Figure 4C**). To explore the mechanisms in the regulation of m7G-associated lncRNA molecular isoforms, we further identified molecular isoform-related differential mRNAs. A total of 174 mRNAs were considered to be regulator genes of molecular subtypes (**Figure 4D**). Interestingly, we found that based on these 174 genes, GC patients could be divided into two regulator subtypes (**Figure S3A**). As with lncRNA molecular typing, there is also significant heterogeneity among different regulatory isoforms (**Figure S3B**), which have survival-indicating ability (**Figure S3C**), and many classical signaling pathways have also been altered between different subtypes, such as Wnt and MAPK. (**Figure S3D**). In addition, the two subtypes have obvious immunological differences and can be used for ICI judgment before treatment (**Figure S4**).

### Calculation of patient risk scores based on m7G-associated lncRNAs

**Figure 5:**
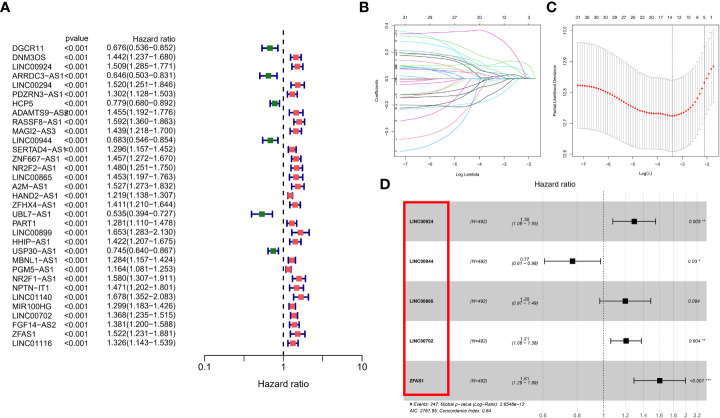
Calculation of patient risk scores based on m7G-associated lncRNAs. **(A)** univariate Cox regression analysis in the GEO cohort **(B, C)** LASSO regression analysis **(D)** risk score formula.

Although the above molecular typing and regulator typing results can predict the survival and functional differences of GC patients, molecular typing is based on the patient population, so it cannot accurately predict the status of each patient. Individual patients were assessed for risk score for clinical application. Considering the large sample size of the GEO cohort, we performed univariate Cox regression analysis in the GEO cohort (**Figure 5A**) to screen out 33 prognostic lncRNAs and then further eliminated redundant lncRNAs by LASSO regression analysis (**Figures 5B, C**). We obtained a risk score formula based on the 5 lncRNAs (**Figure 5D**): (0.2590 × expression level of LINC00924) + (-0.2616 ×expression level of LINC00944) + (0.18349 × expression level of LINC00865) + (0.1899 × expression level of LINC00702) + (0.4736 × expression level of ZFAS1). In addition, we differentiated the TCGA cohort of patients at different risks with the same median risk score. Among them, in the GEO cohort, there were 246 people in the high-risk group and 246 in the low-risk group; in the TCGA cohort, there were 185 people in the high-risk group and 185 people in the low-risk group.

### Prognostic validation of risk scores

**Figure 6:**
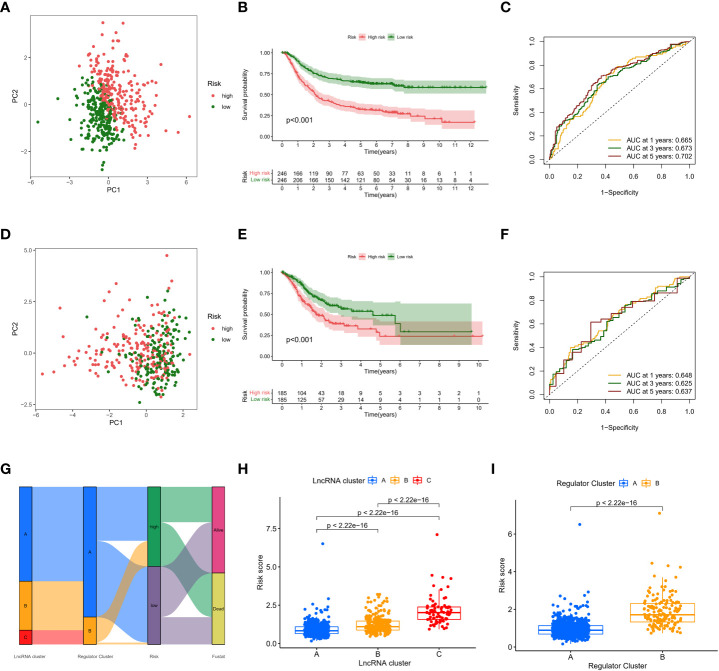
Prognostic validation of risk scores. **(A,D)** PCA showed good dispersion of patients in different cohorts **(B, E)** high-risk patients showed significantly shorter survival times than low-risk patients **(C, F)** AUC curve **(G)** Sankey diagram **(H, I)** boxplots.

**Figure 7:**
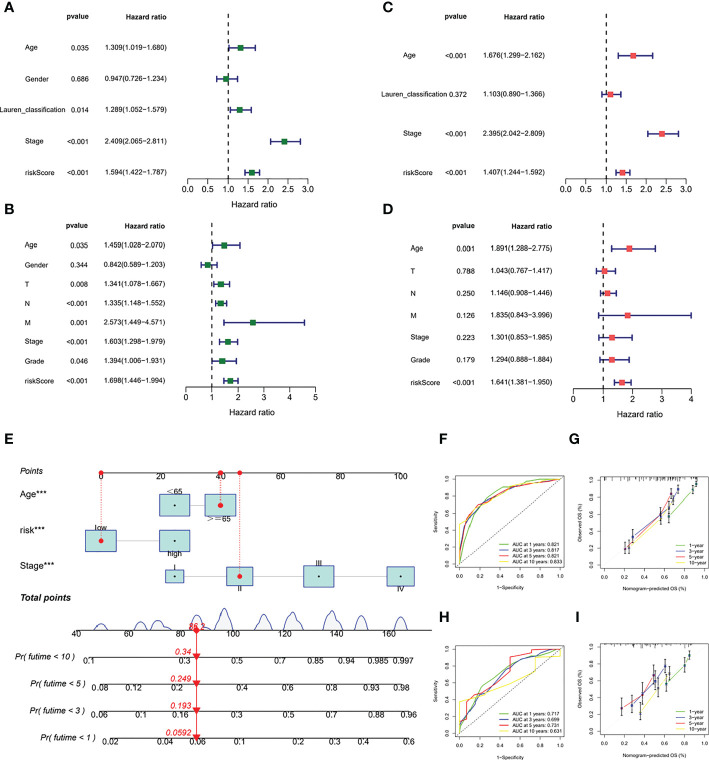
Prognostic validation of risk scores in GC patients. **(A)** Cox regression analysis in GEO cohort **(B)** Cox regression analysis in TCGA cohort **(C, D)** After adjusting other confounding factors, Cox regression analysis in the TCGA and GEO columns **(E)** nomogram **(F, H)** ROC curves **(G, I)** predicted curve.

PCA showed good dispersion of patients in different cohorts (**Figures 6A, D**). In each group, high-risk patients showed significantly shorter survival times than low-risk patients, as shown in **Figures 6B, E**. The AUCs of the GEO cohort at 1, 3, and 5 years were 0.665, 0.673, and 0.702, respectively (**Figure 6C**). Meanwhile, in the TCGA cohort, the predicted AUCs at 1, 2, and 3 years were 0.648, 0.625, and 0.639, respectively (**Figure 6F**). In addition, to explore the link between molecular subtype and risk grouping, we drew a Sankey diagram and found that most of the patients with poor prognosis in the molecular subtype were closely related to those in the high-risk group (**Figure 6G**). In addition, boxplots confirmed our hypothesis that molecular subtype C and regulatory subtype B had higher risk scores (**Figures 6H, I**). To determine whether the risk score is an independent prognostic factor in GC patients, Cox regression analysis was performed by clinical characteristics and risk score. Based on the results of univariate Cox regression analysis, in the TCGA and GEO columns, risk scores were significantly associated with OS (GEO cohort: HR = 1.594 (**Figure 7A**); TCGA cohort: HR = 1.698 (**Figure 7B**). After adjusting for other confounding factors, the risk score was an independent predictor of OS in GC patients (GEO cohort: HR = 1.407 (**Figure 7C**); TCGA cohort: HR = 1.641 (**Figure 7D**). In addition, for more accessible clinical application, we combined staging and risk classifications and drew a nomogram that was intuitively applied to clinical work, as shown in **Figure 7E**. The ROC curves showed that the predictive power of the nomogram for 1-, 3-, 5-, and 10-year survival was significantly improved (**Figures 7F, H**). Again, the predicted curve was close to the standard curve, indicating that the predicted survival rates at 1, 3, 5, and 10 years were closely related to the actual survival rates, as shown in **Figures 7G, I**. In addition, we found that different risk groups were significantly correlated with age, stage, and Lauren classification (**Figure S5**).

### Comprehensive analysis of different risk groups

**Figure 8:**
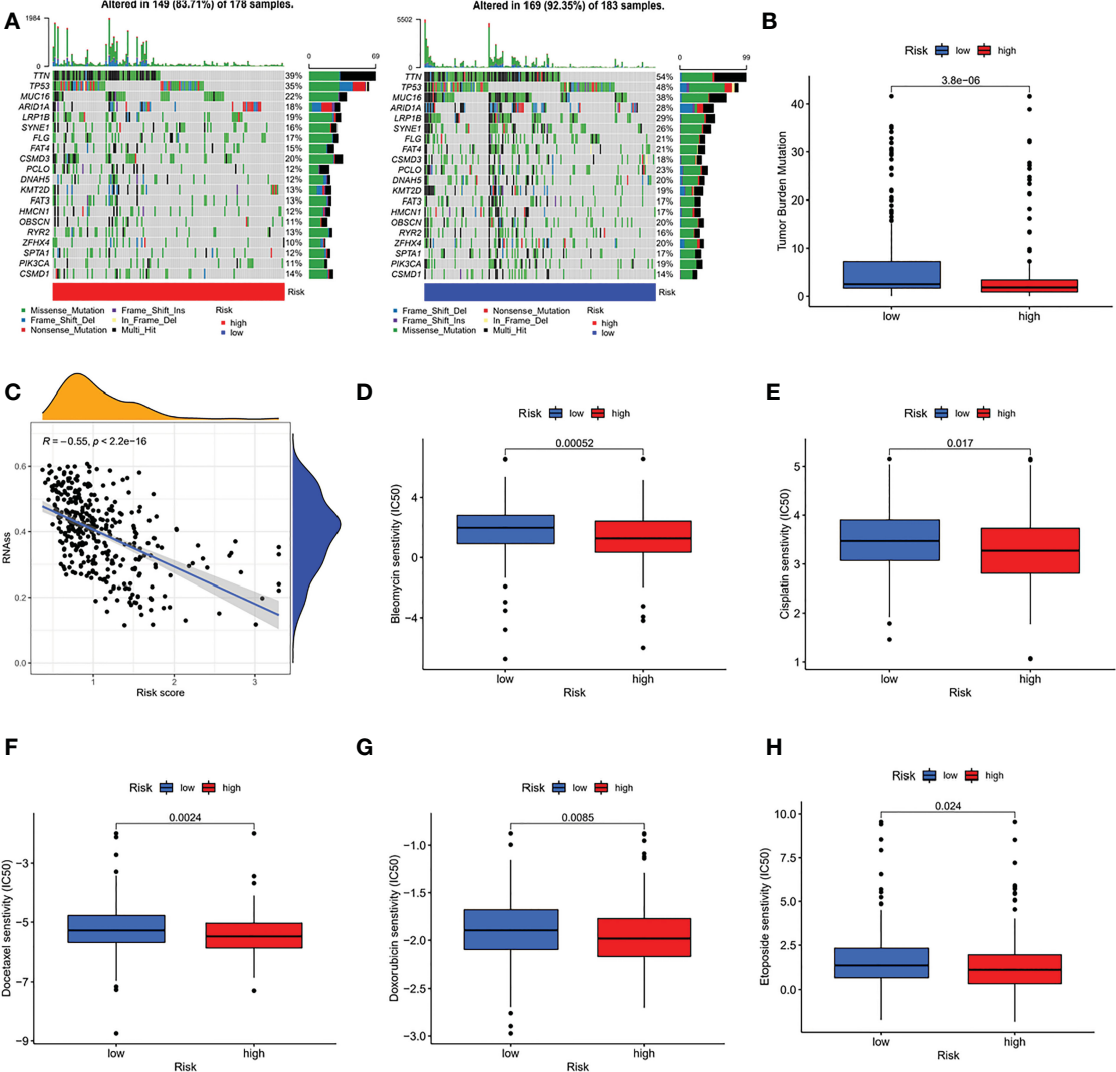
Comprehensive analysis of different risk groups. **(A) **Waterfall plots **(B)** boxplots **(C)** relationship between risk score and the cell stemness index **(D-H)** IC50 of bleomycin **(D)**, cisplatin **(E)**, docetaxel **(F)**, doxorubicin **(G)**, and etoposide **(H)** of high-risk and low-risk groups.

**Figure 9:**
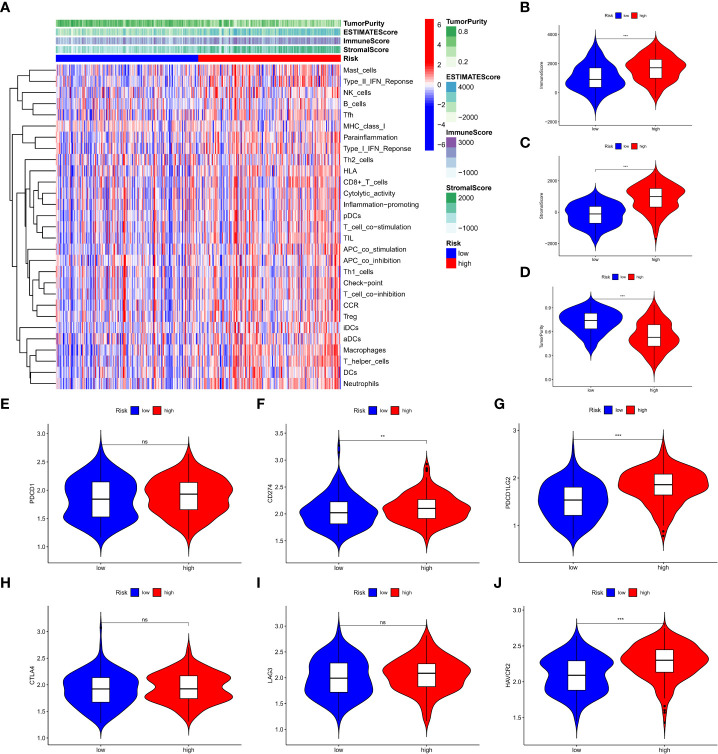
Immune signature analysis of patients in different risk groups. **(A)** degree of infiltration of different groups **(B-D)** assessment of the stromal scores of the three subtypes, immune scores and tumor purity **(E-J)** expression of six immune checkpoint genes. **P < 0.01, ***P < 0.001, ns: no significance.

We further analysed the relationship between the risk score and tumour mutation burden (TMB). Waterfall plots showed that low-risk patients exhibited a wider range of somatic mutation frequencies than high-risk patients (**Figure 8A**), and boxplots showed that high-risk patients had higher TMB scores (**Figure 8B**). Considering the importance of cell stemness maintenance to the survival of tumour patients, we found that with the increase in risk score, the cell stemness index decreased (**Figure 8C**). In addition, we used the pRRophetic algorithm to evaluate the therapeutic effect of chemotherapy drugs. For some classical chemotherapeutic drugs, the IC50 results were encouraging: the high-risk group showed better results than the low-risk group for bleomycin (**Figure 8D**), cisplatin (**Figure 8E**), docetaxel (**Figure 8F**), doxorubicin (**Figure 8G**), and etoposide (**Figure 8H**), which suggests that risk grouping based on the risk score plays a guiding role in chemotherapy. In addition, the GSEA results also suggested that there may be activation states of different pathways in different risk groups, and there may be more activation of apoptosis pathways in high-risk patients (**Figure S6**). We performed an immune signature analysis of patients in different risk groups in the same manner as described above, and patients in the high-risk group showed a higher degree of infiltration (**Figure 9A**). The high-risk group in ESTIMATE showed the highest stromal and immune scores, while tumour purity was lower (**Figures 9B-D**). Finally, we found that the mRNA expression of the six immune checkpoints was also different between the high- and low-risk groups (**Figures 9E-J**), which may suggest that our high-risk group may benefit more from immunotherapy.

### Expression and validation of m7G-related lncRNAs

**Figure 10:**
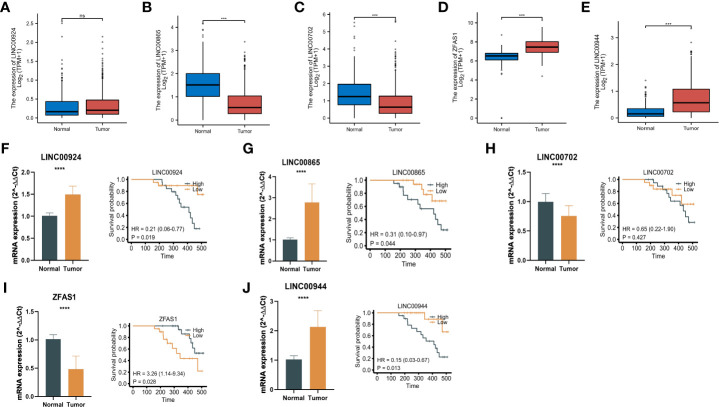
Expression and validation of m7G-related lncRNAs. **(A-E)** The normal gastric gland epithelium sequencing set from the GTEx database and compared it with TCGA-GC. **(F-J)** RT–qPCR results and survival time of patients of the expression of corresponding lncRNAs in 20 pairs of GC and adjacent tissues. *P < 0.05, **P < 0.01, ***P < 0.001, ****P < 0.0001, ns: no significance.

To validate the expression of lncRNAs in m7G-related risk scores, we downloaded the normal gastric gland epithelium sequencing set from the GTEx database and compared it with TCGA-GC (**Figures 10A–E**). In addition, we used RT–qPCR to detect the expression of corresponding lncRNAs in 20 pairs of GC and adjacent tissues in our hospital (**Figures 10F–J**). The results showed that LINC00924, LINC00944, and LINC00865 were highly expressed in tumour tissue, while LINC00702 and ZFAS14 were expressed at low levels in tumour samples. In addition, we analysed the relationship between the five m7G-related lncRNA signatures and patient survival information, and we were pleased to find that the expression level of these m7G-related lncRNAs was correlated with the survival time of patients (**Figures 10F–J**). The overall trend is very consistent with our risk score model. These results strongly validated m7G-related lncRNAs in GCs.

## Discussion

GC continues to be a common malignant cancer worldwide and ranks fifth and fourth in morbidity and mortality, respectively (25). Early GC is mostly asymptomatic or only has mild symptoms, and early GC can be treated by surgical and endoscopic resection (26). Most GC patients are diagnosed at an advanced stage, the effect of surgery and chemotherapy is not good, and the prognosis is poor. Therefore, GC treatment remains a major problem. The mechanism of GC requires further in-depth understanding.

M7G RNA methylation is a modification in which a methyl group is added to the seventh N of the messenger RNA guanine (G) under the action of methyltransferase. m7G modification is one of the most common base modifications in posttranscriptional regulation and is widely distributed in the 5’ cap region of tRNA, rRNA, and eukaryotic mRNA. It plays an important role in maintaining RNA processing metabolism, stability, nuclear export and protein translation (24). In our research, m7G-related lncRNAs and their molecular subtypes were identified, and a total of 123 m7G lncRNAs were identified and divided into 3 subtypes. Among them, type B has the highest degree of infiltration, and the mRNA levels of some immune checkpoints are higher than those of other subtypes, indicating that this subtype may benefit more from immunotherapy. Studies of altered biological processes have shown that, compared to type C, type B is more activated in the following pathways:

KEGG_DRUG_METABOLISM_OTHER_ENZYMES, KEGG_PANTOTHENATE_AND_COA_BIOSYNTHESIS, KEGG_GLYCOSPHINGOLIPID_BIOSYNTHESIS_LACTO_AND_NEOLACTO_SERIE, etc. Drug metabolizing enzymes (DMEs) mainly include phase I enzymes and phase II enzymes, cytochrome P450s (CYPs) are the main phase I enzymes, and glutathione-S-transferases (GSTs), UDP-glucuronosyltransferases (UGTs), and dihydropyridine dehydrogenases (DHPs) are the major phase II enzymes. These enzymes play an important role in the detoxification of exogenous drugs and the metabolism of drugs, and when DMEs are expressed in tumour tissues, drug resistance is mainly caused by metabolizing and inactivating drugs (27). It should be noted that the 123 m7G-related lncRNAs we screened refer to their expression levels correlated with m7G regulators. Whether these m7G-related lncRNAs have an expression regulation mechanism with m7G regulators needs further study.

Next, we obtained a risk score formula based on five lncRNAs, including LINC00924, LINC00944, LINC00865, LINC00702 and ZFAS1, based on the expression of m7G-related lncRNAs. Studies have shown that increased expression of LINC00924 is significantly correlated with decreased overall survival and increased abundance of tumour-infiltrating CD8+ T cells, B cells, macrophages, and NK cells. Immune checkpoint blockers (ICBs) responded poorly to high LINC00924 expression. Furthermore, *via* univariate and multivariate Cox regression analysis, the authors discovered that linc00924-related PEX5L was an independent prognostic factor for GC progression in the CNC (coding–noncoding coexpression) network. That is, LINC00924 expression was associated with poor prognosis and short survival, immune infiltration, and poor response to ICB. LINC00924 may be an immunotherapy target for advanced GC (28). Recent studies have found that LINC00944, a lncRNA related to cancer immunity, is associated with antibiotics, cytokines, interleukins, antigen processing and presentation, natural killer cell cytotoxicity, TCR signaling, cytokines, chemokines and the interleukin receptor pathway (29). Pamela et al. found that the expression of LINC00944 was strongly correlated with immune signaling pathways. Further evaluation of the TCGA-BRCA cohort revealed that LINC00944 expression was positively correlated with tumour-infiltrating T lymphocytes and proapoptotic markers. Furthermore, the results showed that the expression of LINC00944 correlated with age at diagnosis, tumour size, and estrogen and progesterone receptor expression (30). LINC00702 was found to be related to tumour size and tumour metastasis and was significantly downregulated in NSCLC patients. Furthermore, overexpression of LINC00702 *in vitro* and *in vivo* significantly inhibited the proliferation and metastasis of NSCLC cells by inducing apoptosis. Bioinformatics and *in vitro* experiments revealed that LINC00702 functions as a competing endogenous RNA (ceRNA) for miR-510 in NSCLC and upregulates its target gene PTEN. Thus, LINC00702 may become a potential diagnostic biomarker and therapeutic target for NSCLC patients (31). Long noncoding RNAs are key regulators of human disease and prognostic cancer markers, including GC. Using GEO microarray data, Fengqi N et al. comprehensively assessed the transcriptome differences of lncRNAs in GC and identified an oncogenic lncRNA, ZFAS1. ZFAS1 is upregulated in colorectal cancer and hepatocellular carcinoma and functions as an oncogene, and Fengqi N et al. showed that ZFAS1 is also overexpressed in GC, and its elevated levels are associated with poor prognosis and shortened survival. *In vitro* experiments showed that ZFAS1 gene expression inhibited the proliferation and apoptosis of GC cells and inhibited their tumorigenicity *in vivo*. Therefore, ZFAS1 may promote the occurrence of GC (32). In addition, we used RT–qPCR to detect the expression of corresponding lncRNAs in GC and para-carcinoma tissues. The results showed that LINC00924, LINC00944, and LINC00865 were expressed at a high level in tumour tissues, while LINC00702 and ZFAS14 were expressed at low levels in normal samples. The above results suggest that LINC00924, LINC00944, LINC00865, LINC00702 and ZFAS1 play vital roles in the occurrence and development of GC. It is important to note that the results of RT-PCR are not completely consistent with those of BioInfo analysis, which may be due to the limited number of tissue samples for RT-PCR.

Our study also has certain limitations, and the overall conclusions come from bioinformatics analysis of RNA-seq data in the database and lack large-scale clinical validation. At the same time, the main expression of m7G-related lncRNAs in gastric cancer by those cells is not clear, and the specific mechanism of m7G-related lncRNAs affecting the immune response and patient prognosis in gastric cancer also needs to be systematically and deeply experimentally studied. In the future, we will continue to monitor and work hard to advance these issues.

In conclusion, we discovered for the first time that m7G-related lncRNAs play a vital role in the immune microenvironment of GC by regulating immune cell infiltration and immune function in GC tissue in a synergistic manner, and the biological mechanism of their specific functions warrants further study. Our work provides new ideas for the underlying mechanism of GC and immunotherapy.

## Data availability statement

The datasets presented in this study can be found in online repositories. The names of the repository/repositories and accession number(s) can be found in the article/**Supplementary Material**.

## Ethics statement

The studies involving human participants were reviewed and approved by Peking union medical college hospital ethics committee. The patients/participants provided their written informed consent to participate in this study.

## Author contributions

MWM: design, analysis, drafting of the manuscript, complete RT-PCR experiment and critical revision of the manuscript. JL, ZCZ and ZYZ: statistical analysis. WMK: critical revision of the manuscript for important intellectual content, administrative support, obtaining funding, and supervision. All authors contributed to the article and approved the submitted version.
