# Supplementary material for: Integrated analysis from multicentre studies identities m7G-related lncRNA-derived molecular subtypes and risk stratification systems for gastric cancer
Source: Front Immunol. 2023 Mar 2;14:1096488. doi: 10.3389/fimmu.2023.1096488 (PMC10017847; doi:10.3389/fimmu.2023.1096488)
Supplement: Supplementary file 1 [file DataSheet_1.docx]

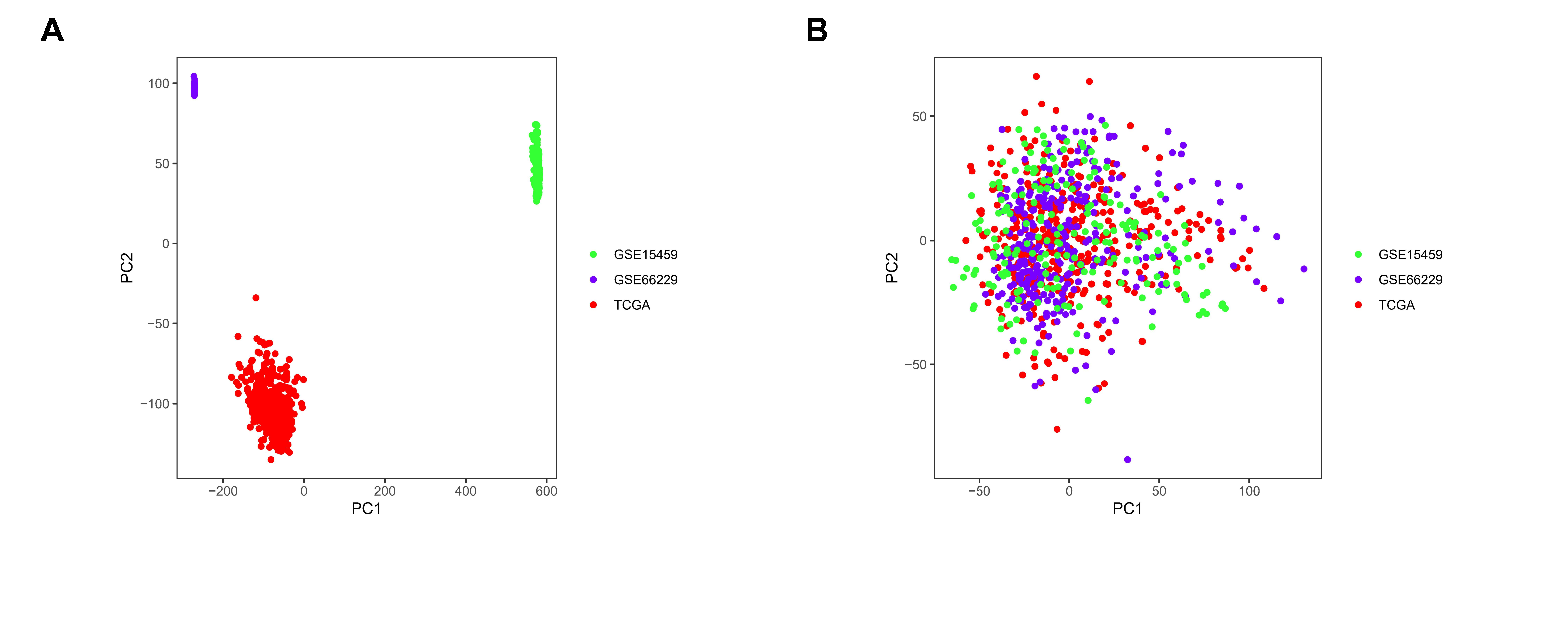


Figure S1 Principal component analysis (PCA) before and after batch correction


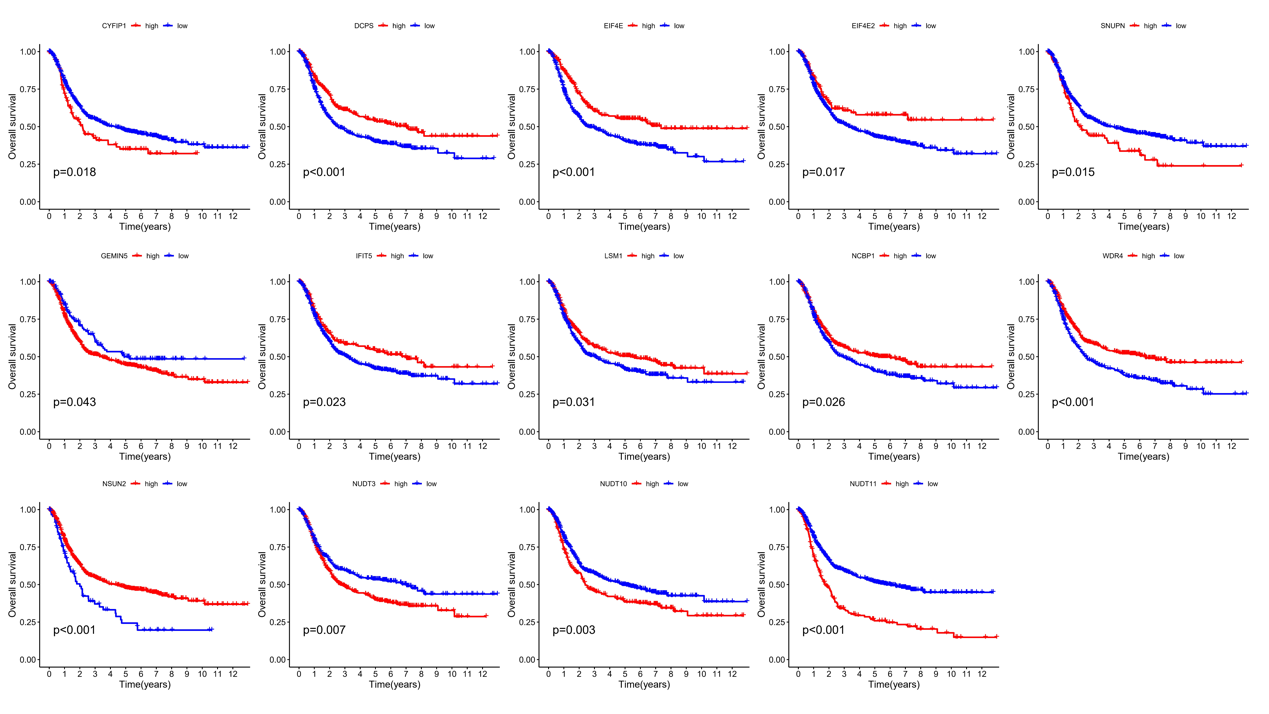


Figure S2 14 regulators as indicators of the prognosis of GC patients


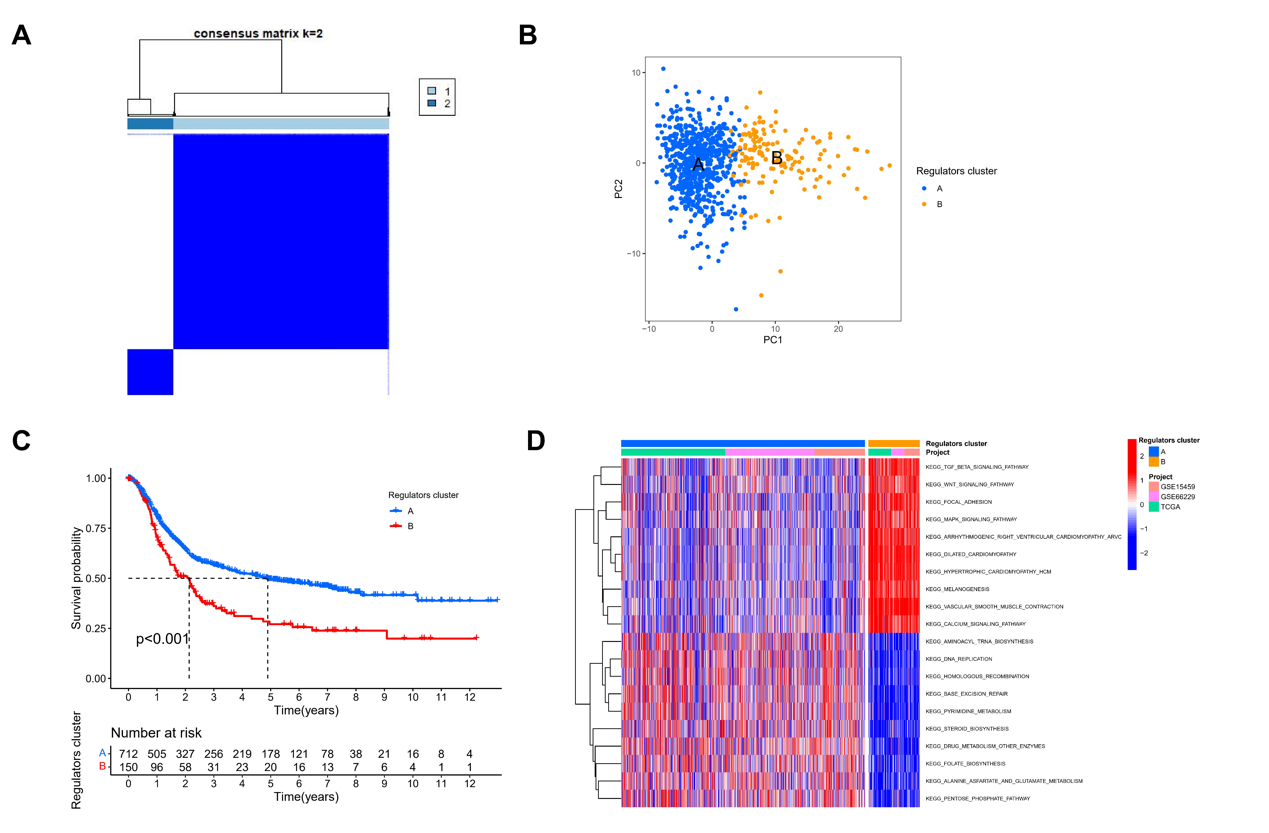


Figure S3 biological functional characterization of m7G-associated lncRNA molecular isoforms


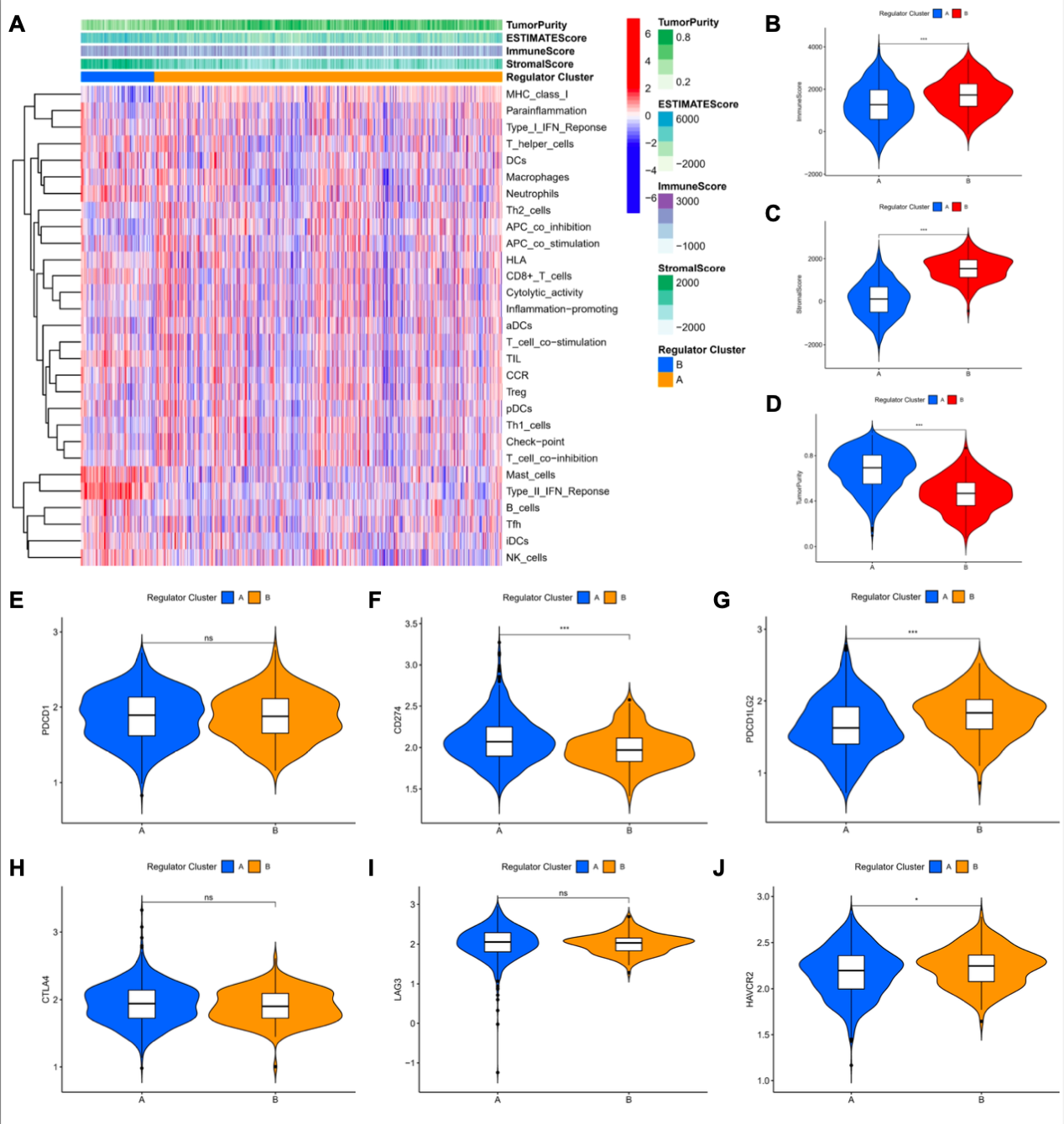


Figure S4 immunological differences of two subtypes


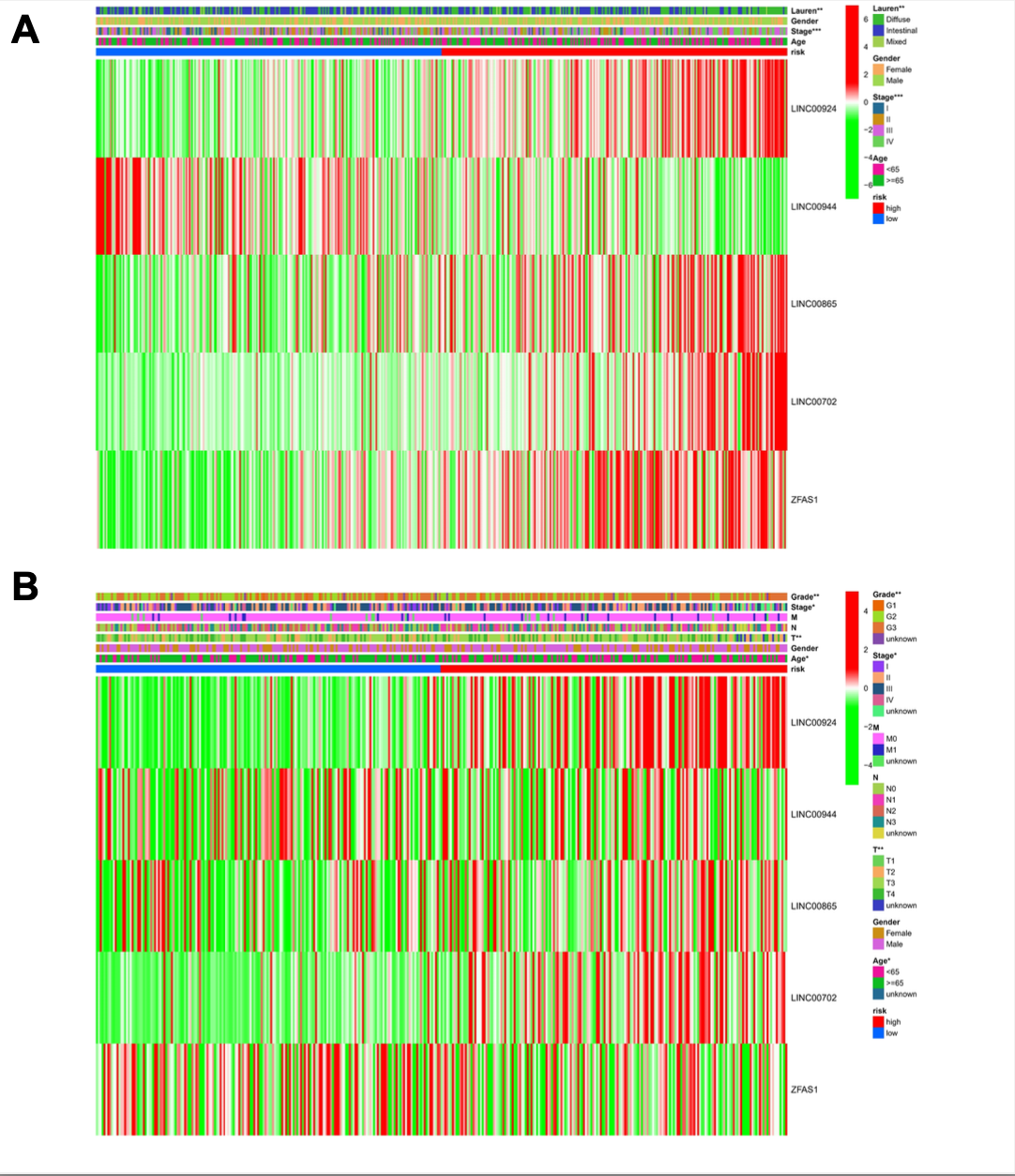


Figure S5 correlation between different risk groups and age, stage, and Lauren


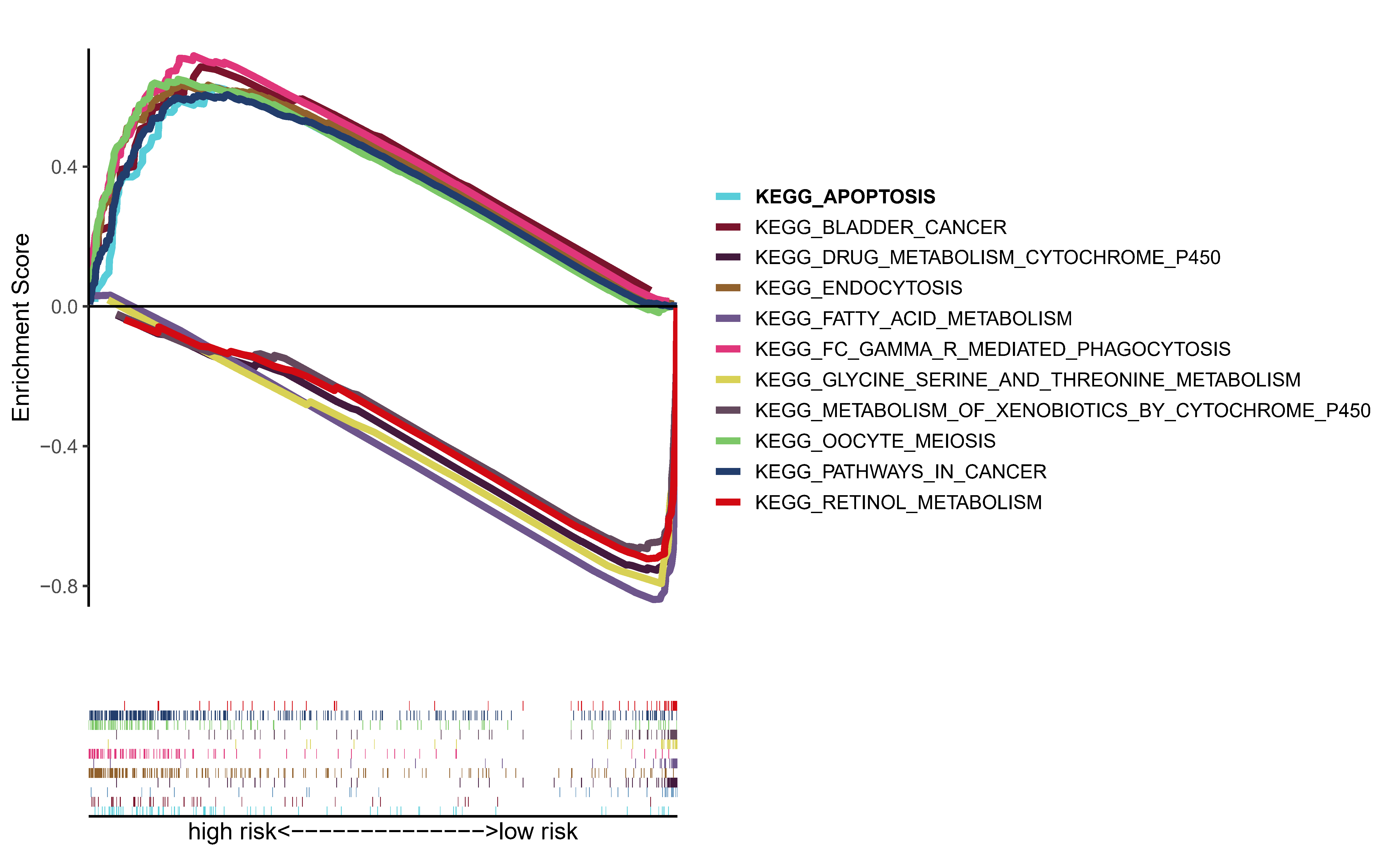


Figure S6 GSEA results of activation states of different pathways in different risk groups

Supplementary File 1 : primer sequences

| Gene | forward primer | reverse primer |
| --- | --- | --- |
| LINC00924 | 5'‐CACTCAAGCAGGATTATTTGTG‐3' | 5'‐TTCAAAGGCAGTTCTGATAGC‐3' |
| LINC00944 | 5'‐CTCCATTCCAGTTTCTGCAC‐3' | 5'‐TCTGATTGAGACCCTGATCC‐3' |
| LINC00865 | 5'‐ACTGGATGTTCCCAGTTCTC‐3' | 5'‐CTTGTACCCAACAGAGTCTC‐3' |
| LINC00702 | 5'‐CACTTCAGAAGACGAAGTGC‐3' | 5'‐ATTCACCGGGAGGAATCTC‐3' |
| ZFAS1 | 5'‐GAAGAGGGAGTCACCACTG‐3' | 5'‐CCAACAATAAACTCGTCAGGAG‐3' |
| GAPDH | 5'‐ACAACTTTGGTATCGTGGAAGG‐3' | 5'‐GCCATCACGCCACAGTTTC‐3' |
